# Haematuria As the Initial Manifestation of Bladder Plasmablastic Lymphoma: A Report of a Rare Case and Literature Review

**DOI:** 10.7759/cureus.105696

**Published:** 2026-03-23

**Authors:** Fares A Idrees, Jalaja Mary George

**Affiliations:** 1 Urology, Calderdale and Huddersfield NHS Foundation Trust, Halifax, GBR; 2 Histopathology, Calderdale and Huddersfield NHS Foundation Trust, Halifax, GBR

**Keywords:** bladder lymphoma, case report, diffuse large b-cell lymphoma, extranodal lymphoma, hematuria, plasmablastic lymphoma

## Abstract

Plasmablastic lymphoma (PBL) is an aggressive and uncommon variant of diffuse large B-cell lymphoma, most frequently associated with immunocompromised states. Although extranodal disease is characteristic, involvement of the urinary bladder is exceptionally rare and may mimic primary urothelial malignancy both clinically and histologically.

We report the case of a 62-year-old man with a previous history of high-grade diffuse large B-cell lymphoma in remission who presented with acute gross haematuria. Imaging demonstrated bladder wall thickening with associated hydronephrosis. Transurethral resection of the bladder lesion, followed by detailed histopathological and immunohistochemical analysis, confirmed plasmablastic large B-cell lymphoma involving the bladder.

This case underscores the importance of maintaining a broad differential diagnosis in patients presenting with haematuria, particularly those with a prior history of haematologic malignancy. A review of the literature highlights the rarity of bladder involvement and the diagnostic challenges associated with this entity.

## Introduction

Plasmablastic lymphoma (PBL) is a rare and highly aggressive subtype of non-Hodgkin lymphoma characterised by plasmacytic differentiation and a high proliferative index; it accounts for less than 1% of all non-Hodgkin lymphomas and approximately 2% to 3% of diffuse large B-cell lymphomas [1,2]. It was initially described in patients with human immunodeficiency virus (HIV) infection [1] but has since been reported in immunocompetent individuals and in patients with prior haematologic malignancies [2]. PBL demonstrates a marked male predominance with a reported male-to-female ratio of approximately 3:1 [2].

Extranodal involvement is present in the majority of cases, occurring in approximately 70% to 80% of patients, most frequently affecting the oral cavity and gastrointestinal tract [2]. Genitourinary involvement is distinctly uncommon, and bladder involvement in particular has been reported only in isolated cases. Because of its morphologic overlap with poorly differentiated urothelial carcinoma and plasmacytoid carcinoma, bladder PBL represents a significant diagnostic challenge [2].

We describe a rare case of bladder PBL presenting with gross haematuria in a patient previously treated for diffuse large B-cell lymphoma, followed by a focused review of the literature.

## Case presentation

A 62-year-old man presented to the emergency department on December 26, 2024, with a one-day history of frank haematuria. He denied trauma, dysuria, or constitutional symptoms.

His medical history was notable for high-grade diffuse large B-cell lymphoma diagnosed in 2018, treated with six cycles of rituximab, cyclophosphamide, doxorubicin, vincristine, and prednisone (R-CHOP) chemotherapy, resulting in remission. He had residual chemotherapy-induced peripheral neuropathy. Other comorbidities included left total hip replacement (2021), biliary stenting for obstruction (2018), and a transient monoclonal gammopathy of undetermined significance that had resolved.

**Figure 1:**
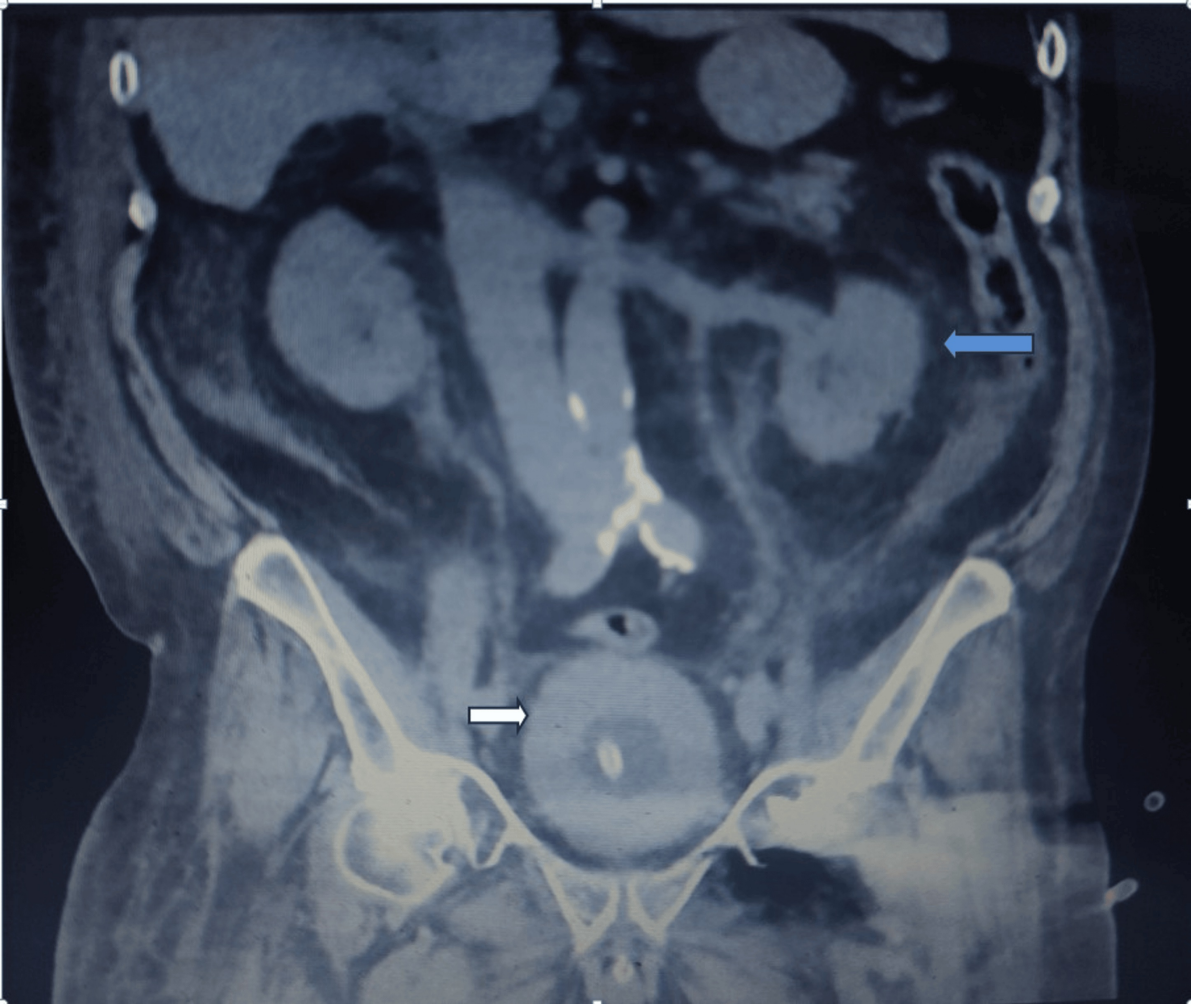
CT of the kidney, ureters and bladder showing marked bladder wall thickening (white arrow) and left hydronephrosis (blue arrow)

Initial laboratory investigations demonstrated anaemia and impaired renal function. Computed tomography of the kidneys, ureters, and bladder (CT KUB) revealed bladder wall thickening with marked left-sided hydronephrosis (Figure 1). Attempted retrograde ureteric stenting was unsuccessful due to involvement of the left ureteric orifice by the bladder lesion.

The patient underwent bladder washout and transurethral resection of bladder tumour on two occasions because of persistent bleeding and clot retention. A left antegrade nephrostomy and ureteric stent were subsequently placed by interventional radiology.

**Figure 2:**
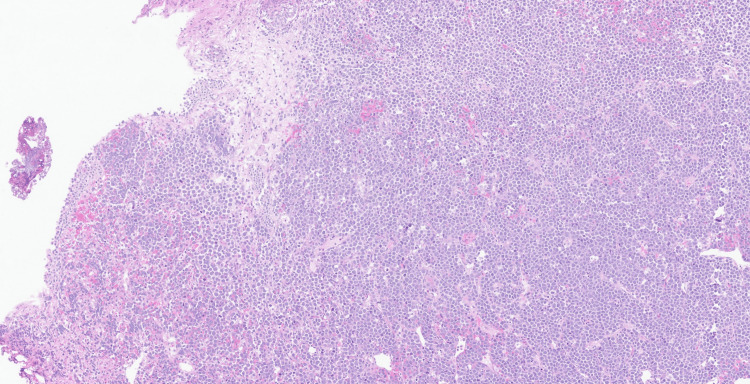
Haematoxylin and eosin-stained section (x10) Diffuse infiltration of the bladder wall by a highly cellular neoplasm composed of sheets of large, poorly cohesive atypical cells is noted.

**Figure 3:**
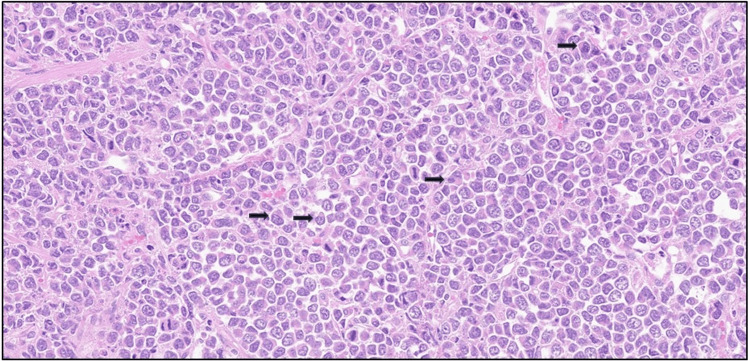
Haematoxylin and eosin-stained section (×40) Tumour cells with eccentrically placed nuclei (black arrow), prominent nucleoli, and brisk mitotic activity are visualised.

Microscopic examination demonstrated an infiltrating high-grade malignant neoplasm involving the lamina propria and extensively infiltrating the muscularis propria. The tumour was composed of diffuse sheets of large, poorly cohesive atypical cells with a high nuclear-to-cytoplasmic ratio, vesicular chromatin, conspicuous nucleoli, and moderate pleomorphism (Figure 2). Numerous mitotic figures and apoptotic bodies were present, with areas of tumour necrosis (Figure 3). The overlying urothelium was largely denuded; where preserved, it showed no evidence of carcinoma in situ. No definite lymphovascular invasion was seen.

**Figure 4:**
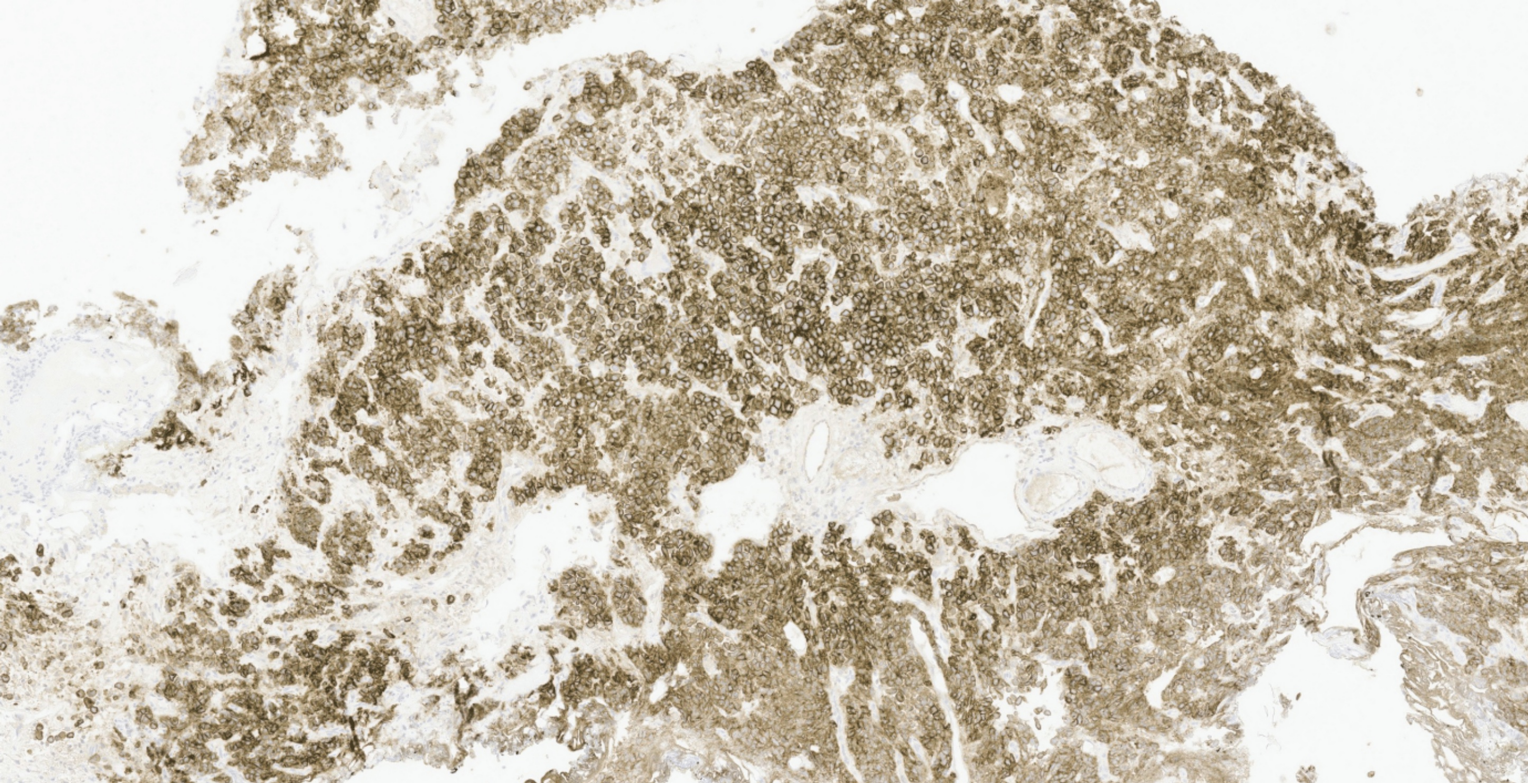
Immunohistochemistry (×10) showing strong, diffuse membranous CD30 staining.

**Figure 5:**
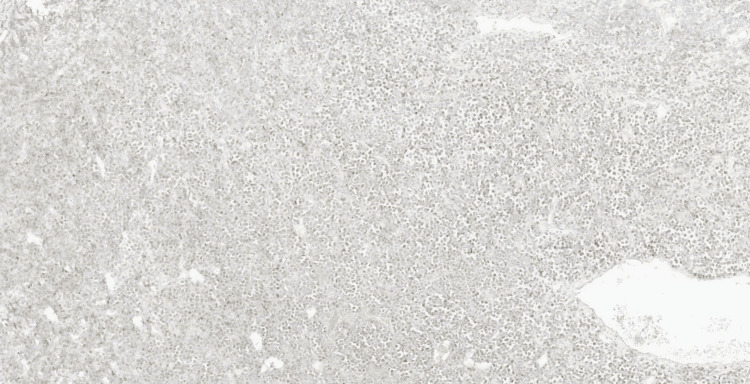
Immunohistochemistry (×10) showing weak, diffuse nuclear GATA3 staining.

**Figure 6:**
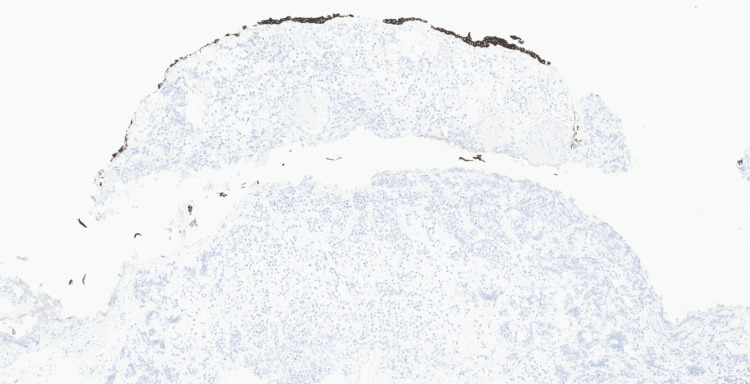
Immunohistochemistry (×10) showing MNF116 negative in tumour cells with retained strong cytoplasmic staining in the overlying urothelium.

**Figure 7:**
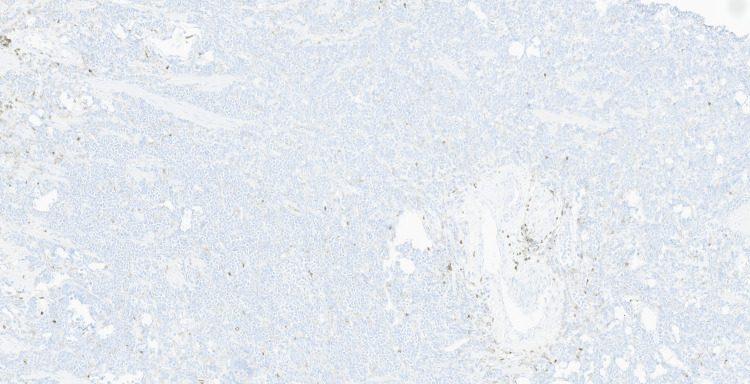
Immunohistochemistry (×10); absence of LCA expression is noted.

**Figure 8:**
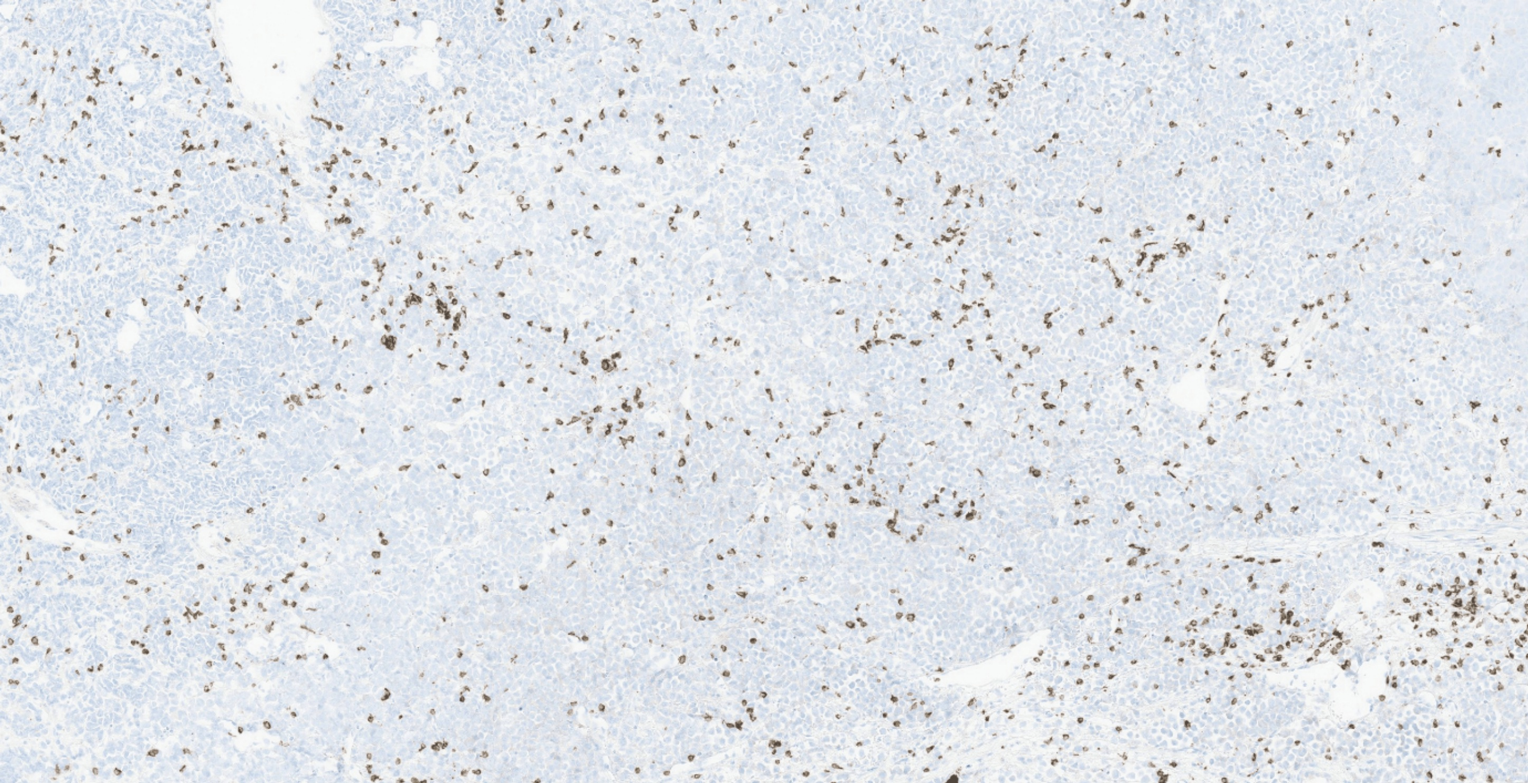
Immunohistochemistry (×10); absence of CD3 expression is noted.

**Figure 9:**
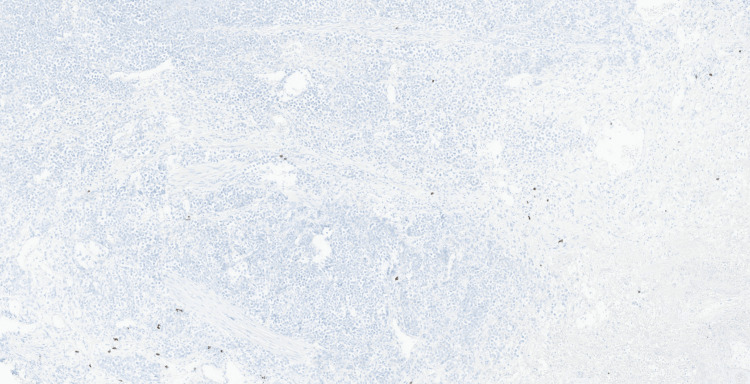
Immunohistochemistry (×10); absence of CD20 expression is noted.

**Figure 10:**
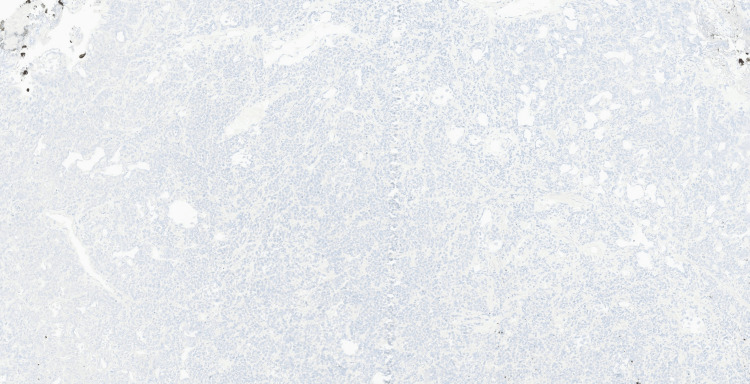
Immunohistochemistry (×10); absence of AE1/AE3 staining is noted.

**Figure 11:**
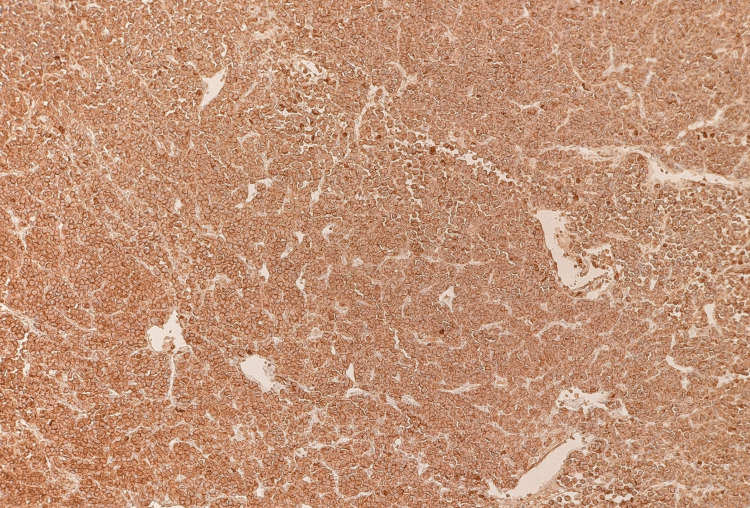
Immunohistochemistry (×10) showing strong, diffuse cytoplasmic CD138 staining.

Initial immunohistochemical studies performed demonstrated strong CD30 expression in the neoplastic cells (Figure 4), with faint and patchy GATA3 positivity (Figure 5). The tumour cells were negative for broad-spectrum cytokeratins (AE1/AE3, MNF116), CK7, CK20, leukocyte common antigen CD45, CD3, CD20, PLAP, synaptophysin, chromogranin, E-cadherin, S100, and HMB45, excluding epithelial, melanocytic, neuroendocrine, and conventional urothelial differentiation (Figures 6-10). A CD30-positive high-grade lymphoma was initially considered. In view of focal plasmacytoid morphology, CD138 immunohistochemistry was subsequently performed and showed strong and diffuse positivity, raising the possibility of plasmablastic lymphoma (Figure 11). Given the unusual morphology, immunophenotype, and the patient’s prior history of diffuse large B-cell lymphoma, the specimen was referred to the Haematology Malignancy Diagnostic Service (HMDS), Leeds, England, for further characterisation. 

At the reference centre review, the extended immunohistochemistry demonstrated strong expression of CD138, CD30, and IRF4/MUM1 with a very high proliferative index (Ki-67 approximately 100%). The tumour cells were negative for CD20, PAX5, CD79a, CD10, BCL2, BCL6, Cyclin D1, ALK-1, and T-cell markers. Light chain studies demonstrated kappa restriction.

Ancillary molecular studies confirmed B-cell clonality with clonal immunoglobulin heavy chain rearrangements (FR3 and incomplete DH-JH strategies). Epstein-Barr virus in situ hybridisation was negative.

The combined histomorphological, immunophenotypic, and molecular findings were diagnostic of plasmablastic large B-cell lymphoma involving the urinary bladder.

The principal differential diagnoses considered included extramedullary plasmacytoma/plasma cell myeloma with blastoid morphology and poorly differentiated urothelial carcinoma, plasmacytoid variant; these were excluded on immunohistochemical and molecular grounds. The lesion was regarded as a new aggressive B-cell lymphoid neoplasm, with an uncertain relationship to the patient’s prior history of diffuse large B-cell lymphoma.

After histological confirmation of PBL, the patient was referred to haematology for further evaluation and planning of systemic therapy. Prior to initiation of lymphoma-directed treatment, the patient developed an acute cardiovascular event and died during the same admission. Consequently, no oncological treatment was administered, and treatment response could not be evaluated.

## Discussion

PBL is a rare and aggressive subtype of non-Hodgkin lymphoma first described in association with HIV infection and most commonly involving the oral cavity [1,2]. Reported genitourinary cases have occurred predominantly in immunocompromised patients, although cases in immunocompetent individuals have also been described [2,3].

Extranodal disease is frequent, but genitourinary involvement is distinctly uncommon [4]. Only a limited number of cases involving the urinary tract have been reported, including involvement of the ureter, kidney, and bladder [5,6]. Yamamoto et al. described a ureteric PBL presenting as a polypoid mass in an HIV-positive patient [5], while Jafarizade et al. reported bladder involvement associated with light-chain cast nephropathy [6]. These cases highlight the variability in clinical presentation and the rarity of urinary tract localisation.

Histologically, PBL may closely resemble poorly differentiated urothelial carcinoma or plasmacytoid neoplasms, making diagnosis challenging, particularly in limited biopsy specimens. Immunohistochemistry demonstrating plasma cell markers such as CD138 and MUM1, with absent or weak expression of CD20, is essential for accurate diagnosis [1,2].

Because of the small number of reported genitourinary cases and the heterogeneity of available clinical data, optimal management remains unclear. Most patients described in the literature received systemic chemotherapy, but the overall prognosis is poor, particularly in patients with advanced disease or significant comorbidity [2,3]. Our case adds to the limited literature describing bladder involvement and further emphasises the diagnostic difficulty and aggressive clinical behaviour of this rare entity.

## Conclusions

Primary bladder PBL is an exceptionally rare but highly aggressive malignancy with a recognised epidemiological pattern, occurring more commonly in middle-aged to older males and frequently associated with immunosuppression, particularly HIV infection. These risk factors are associated with poorer overall prognosis and limited response to therapy. Bladder involvement may present with nonspecific urological symptoms such as haematuria and can mimic urothelial carcinoma, making histological confirmation essential. In patients with a history of lymphoma or immunosuppression, bladder lesions should raise suspicion for haematologic malignancy. Early diagnosis and multidisciplinary management are critical, although outcomes remain poor, as illustrated in our case.
